# Root samples provide early and improved detection of *Candidatus* Liberibacter asiaticus in *Citrus*

**DOI:** 10.1038/s41598-020-74093-x

**Published:** 2020-10-12

**Authors:** W. Evan Braswell, Jong-Won Park, Philip A. Stansly, Barry Craig Kostyk, Eliezer S. Louzada, John V. da Graça, Madhurababu Kunta

**Affiliations:** 1 Mission Laboratory, USDA APHIS PPQ S&T, Edinburg, TX 78541 USA; 2 Texas A&M University-Kingsville Citrus Center, 312 N. International Blvd., Weslaco, TX 78599 USA; 3 Southwest Florida Research and Education Center, University of Florida-IFAS, Immokalee, FL 34142 USA

**Keywords:** Plant ecology, Plant stress responses

## Abstract

Huanglongbing (HLB), or Citrus Greening, is one of the most devastating diseases affecting agriculture today. Widespread throughout *Citrus* growing regions of the world, it has had severe economic consequences in all areas it has invaded. With no treatment available, management strategies focus on suppression and containment. Effective use of these costly control strategies relies on rapid and accurate identification of infected plants. Unfortunately, symptoms of the disease are slow to develop and indistinct from symptoms of other biotic/abiotic stressors. As a result, diagnosticians have focused on detecting the pathogen, *Candidatus* Liberibacter asiaticus, by DNA-based detection strategies utilizing leaf midribs for sampling. Recent work has shown that fibrous root decline occurs in HLB-affected trees before symptom development among leaves. Moreover, the pathogen, *Ca.* Liberibacter asiaticus, has been shown to be more evenly distributed within roots than within the canopy. Motivated by these observations, a longitudinal study of young asymptomatic trees was established to observe the spread of disease through time and test the relative effectiveness of leaf- and root-based detection strategies. Detection of the pathogen occurred earlier, more consistently, and more often in root samples than in leaf samples. Moreover, little influence of geography or host variety was found on the probability of detection.

## Introduction

Huanglongbing (HLB) disease is ravaging the *Citrus* industry around the world. Known as Citrus Greening in many areas, due to the incomplete ripening of fruit in affected trees, the disease has been detected in over 58 countries in the tropical and subtropical regions of Africa, Asia, Oceania, Americas and the Caribbean^1^. By reducing fruit quality, fruit yield, and tree lifespan^2–5^ in affected trees, HLB is causing severe economic losses.

Within the United States, HLB was first detected in Florida in 2005^6^, a year after the detection of the disease in Brazil^7^. Over the subsequent ten growing seasons (i.e., from 2006–2007 to 2015–2016), Florida is estimated to have lost $4.6 billion in industry output, $2.77 billion in value added impacts, $1.8 billion in labor income, and 34,124 jobs as a result of HLB^8^. The disease has since spread to most *Citrus* producing states with detections in Louisiana and Mississippi in 2008, South Carolina and Georgia in 2009, California and Texas in 2012; and Alabama in 2017^1,9,10^.

Although Koch’s postulates have not been met, due to the unculturable nature of the bacteria, extensive evidence supports the conclusion that *Candidatus* Liberibacter asiaticus (CLas)^11^, *Ca.* Liberibacter africanus (CLaf)^11^, and *Ca.* Liberibacter americanus (CLam)^12^ are the causative agents of Huanglongbing disease. These bacteria are vector-borne and phloem-limited within the plant. Inoculation occurs during feeding by one of two highly mobile psyllid species, *Diaphorina citri* Kuwayama (Asian citrus psyllid) for CLas and CLam and *Trioza erytreae* Del Guercio (African citrus triozid) for CLaf^13–15^. Both vectors prefer to feed and oviposit on newly developing flushes of young leaves^14,16–19^. As a result, young flush represents the site of pathogen inoculation^20^.

Most work on HLB has focused on CLas as this species is the most widespread, reaching most *Citrus* producing areas in Asia, Africa, and the Americas^3^, and appears to cause more severe symptoms^21,22^. CLas invades plants during feeding by *D. citri*^13^. Following inoculation, disease detection may take between 3 and 6 months for a greenhouse tree and more than a year for field trees^17,23–25^.

Although inoculation occurs in young growing leaves, it is clear that CLas and CLam move through the plant vascular system^21,22,26–30^. However, disease progression within the plant after inoculation is poorly understood. In an effort to identify the plant organ most likely to provide reliable sources of CLas for real-time PCR-based detection, Li et al.^28^ sampled from petioles, leaf mid-ribs, leaf blade, green stem bark, mature bark, roots, and fruit from symptomatic plants. They found the highest bacterial titers within petioles and leaf mid-ribs. Similarly, significantly higher CLas titers were found in peduncle, columella, and leaf midribs compared to seeds, young shoots, flower buds, flowers, and bark. These results led to a focus on foliar tissue for diagnostic analysis that has provided years of successfully implemented diagnostics.

Although detection of CLas from symptomatic leaves is straightforward^30^, symptoms can be slow to develop with this disease^23^. Methods for early detection have been called for by growers and systematic reviews of the disease management process^31,32^. While PCR-based detection of CLas in leaf midribs are highly sensitive, the distribution of pathogen within the tree canopy is patchy^27,33^. Efforts to overcome the potential for false negative diagnostic tests due to the uneven distribution of CLas in the tree canopy have focused on pooling midribs from multiple leaves^31^, thereby improving the probability of including infected tissue in the diagnostic sample.

The earliest evidence that alternative sampling locations may be profitable, came from Graham et al.^34^ and Johnson et al.^29^. Their data showed that the earliest symptom of HLB was found in the roots. Following that observation, Louzada et al.^33^ demonstrated that CLas was consistently and evenly distributed throughout fibrous roots of infected trees. This result suggested that the use of root samples for diagnostics could alleviate difficulties caused by the patchy distribution of CLas within the canopy. Park et al.^35^ then demonstrated that CLas can be detected in the roots of asymptomatic trees. Despite these advances, each of these studies^27–29,33–36^ were based on limited sample sizes and could not evaluate the probabilities of detection using different samples or determine which sample provided earliest detection. Here, we report on the first large-scale, longitudinal study of CLas detection to address these questions.

## Materials and methods

### Field sites and sample collection

To evaluate the relative utility of sampling roots and leaves for the detection of CLas, we established a longitudinal study to track the fate of individual trees in Florida and Texas. We searched for young *Citrus* trees (4 to 5 years old) lacking visible symptoms of HLB but that had a high likelihood of becoming infected during the course of the study. A ca. 4-hectare block of young sweet orange (Hamlin) trees on Cleopatra mandarin rootstock was identified in an orchard in Immokalee, FL. This block of sweet orange trees was surrounded by ca. 31.5 hectares of 10–15-year-old mature trees with a high incidence of HLB. A ca. 2-hectare block of young grapefruit trees on sour orange rootstock in Donna, TX was also identified. This block was bordered on the north and south by ca. 33 acres of 10–15-year-old mature trees, where routine HLB survey showed a gradual increase of HLB incidence (data not shown).

**Figure 1:**
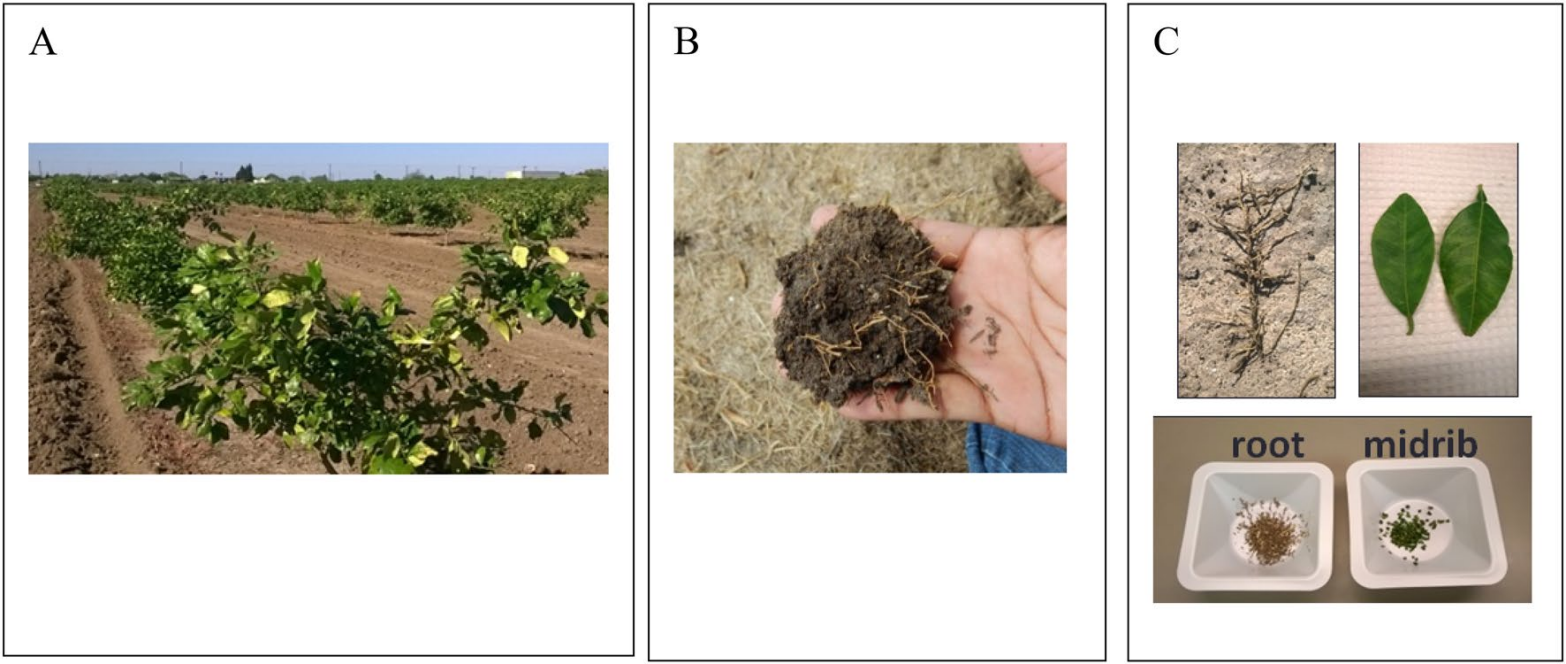
Images depicting *Citrus* leaf and root samples typical of those used in this study. (**A**) Grapefruit trees in the field in Texas. (**B**) Fibrous roots as collected from the field. (**C**) Fibrous root cleaned of soil (upper left), symptomatic leaf (upper right), and root and midrib samples chopped for DNA extraction (bottom).

The sweet orange and grapefruit trees in these two blocks were intensively surveyed for visible symptoms of HLB (Fig. 1a,c). Those trees that lacked visible symptoms, 99 sweet orange trees in Florida and 112 grapefruit trees in Texas, were selected for continued study. Leaf samples were collected and processed according to USDA protocol^37^. Eight to ten leaves were collected from throughout the canopy of each tree with preference given to symptomatic leaves when present. Root samples were collected from two locations for each tree, approximately two feet from the trunk and perpendicular to the row direction on opposite sides of the tree, by digging one to five inches deep into the soil with a small hand trowel (Fig. 1b). Approximately, one to two grams of small fibrous roots (≤ 1 mm in diameter, Fig. 1c) were collected from larger feeder roots and stored in paper bags for transport to the laboratory. Leaf and root samples were collected from each tree on a monthly basis from November 2015 through September 2016 in Florida (excluding December), and from January through November 2016 in Texas. In total, 1980 samples (990 leaf and 990 root samples) in Florida and 2464 samples (1232 leaf and 1232 root samples) were collected and examined in the study.

### Nucleic acid extraction and real-time PCR analysis

In the laboratory, leaf petioles and midribs were separated from leaf blades and chopped, each with a new sterile razor blade, to 1–2 mm in length to aid maceration prior to DNA extraction (Fig. 1c). Two hundred milligrams of pooled, chopped petiole and mid-rib tissue was used for DNA extraction with the Qiagen BioSprint 96 workstation and BioSprint 96 DNA Plant kit following the manufacturer protocol (Qiagen, Hilden, Germany). The leaf DNA fraction was eluted in 200 µl of the kit elution buffer. Root samples were air dried for 24 h at 25 °C, thoroughly dusted to remove excess soil (Fig. 1c) and chopped, each with a new sterile razor blade, to 1–2 mm in length to aid maceration prior to DNA extraction (Fig. 1c). Chopped roots from a single tree were mixed, and a 150 mg subsample was used for DNA extraction with the DNeasy PowerPlant Pro HTP96 kit (Qiagen, Hilden, Germany). The root DNA fraction was eluted in 100 µl of the kit elution buffer. Potential PCR inhibitors were removed from the root DNA fraction with the OneStep-96 PCR inhibitor removal kit (Zymo Research, Irvine, CA, USA), after which the root DNA fraction was diluted 1:1 with 100 µl of nuclease-free water (Ambion, Austin, TX, USA) as described by Park et al.^35^. DNA concentration was measured on NanoDrop 2000 (Thermo Scientific, Waltham, MA, USA).

Extracted DNA from leaf and root samples were tested for the presence of CLas using real-time PCR protocols specific to the organ from which the DNA originated as described below. Leaf DNA samples were tested using real-time PCR with the HLBaspr primer/probe system^30^ to detect a region of the CLas 16S rDNA. This marker system provides highly sensitive detection from leaf-based DNA extracts but binds to and amplifies non-specific targets found within root-based DNA extracts^38^. Thus, to test root samples for the presence of CLas, we employed the TXCChlb primer/probe system^35^, which targets the same gene using primers that avoid off-target amplification. Nuclease-free water and a plasmid DNA that contains CLas 16s rDNA target were used as a negative and positive control for real-time PCR reaction, respectively.

Detection of CLas was defined as acquisition of a real-time PCR quantification cycle (Cq) value less than 37 following USDA protocol^37^. Reactions that produced a Cq of 37 or greater were scored as negative. Upon identification of at least one sample from either organ (root or leaf) producing a detection of CLas, trees were classified as “diagnosed" with HLB. Cq values were also used to estimate bacterial titer (i.e., Log_10_(Genome Equivalents) per gram of sample) using the amplification efficiency equations published by Li et al.^39^ for leaf and Park et al.^35^ for root. The estimated detection limit of both real-time PCR methods with a Cq cutoff < 37 is approx. 10^2^ copies of a plasmid DNA containing a partial CLas 16s rDNA (data not shown). After developing the real-time PCR protocol for CLas detection in fibrous roots using TXCChlb primer–probe set^35^, we have extensively evaluated the consistency of CLas detection between TXCChlb- and HLBaspr-based assay systems^39^ against leaf DNA fractions prepared from Citrus trees growing in Texas.

### Statistical analyses

The influence of sample type, location, and month on bacterial titer (as measured by number of genome equivalents) from trees diagnosed with HLB was determined using factorial Analysis of Variance (ANOVA). Since not all samples from a tree diagnosed with HLB produce positive detections, we tested for the influence of negative real-time PCR reactions with a second factorial ANOVA using only those real-time PCRs that produced positive CLas detections.

Contingency analysis was used to evaluate differences in the frequency of tree diagnosis by each sample type (i.e., the sample in which CLas was first detected). Similarly, we compared all samples (i.e., regardless of detection order) in which CLas was detected using contingency analysis. To evaluate the consistency of detection (i.e., detections in repeated samples through time) for each sample type, we compared the average number of detections per tree between leaf and root samples using t-tests. Since earlier detections in one sample type over another could bias the number of detections, we compared the proportion of detections in each sample type following initial detection within that organ using contingency analysis. To evaluate whether use of leaf or root samples resulted in earlier detection, we used contingency analysis to compare the number of trees diagnosed (i.e., first detected) by each sample type.

Parametric survival analysis was used to estimate the probabilities of diagnosis and of detection from each sample type and for each location. We scored diagnosis of trees and detection of CLas within samples as events and measured the time until each event occurred. Trees that lacked detection within either sample type by the end of the study were censored. Sample collection dates in Florida and Texas did not align so for this analysis we used sample period, rather than the month of sample collection, to allow joint analysis of Florida and Texas trees (however, independent analyses produced similar results). Goodness of fit testing along with corrected Akaike Information Criterion (AICc) and Bayesian Information Criterion (BIC) were used to choose the best fit distribution to model the time until detection for each tree. We used parametric survival analysis to compare the time until detection for each tree between leaf- and root-based samples and to evaluate how the probability of detection changed through time. We used goodness-of-fit tests to evaluate the influence of sample type, location, and their interaction on time until detection. Finally, we used t-tests to evaluate whether there was a significant time savings from testing one sample type over the other. All data was analyzed in JMP ver. 13.1.0 (SAS Institute, Cary, NC, 1989–2019).

## Results

### Pattern of CLas detection

**Figure 2:**
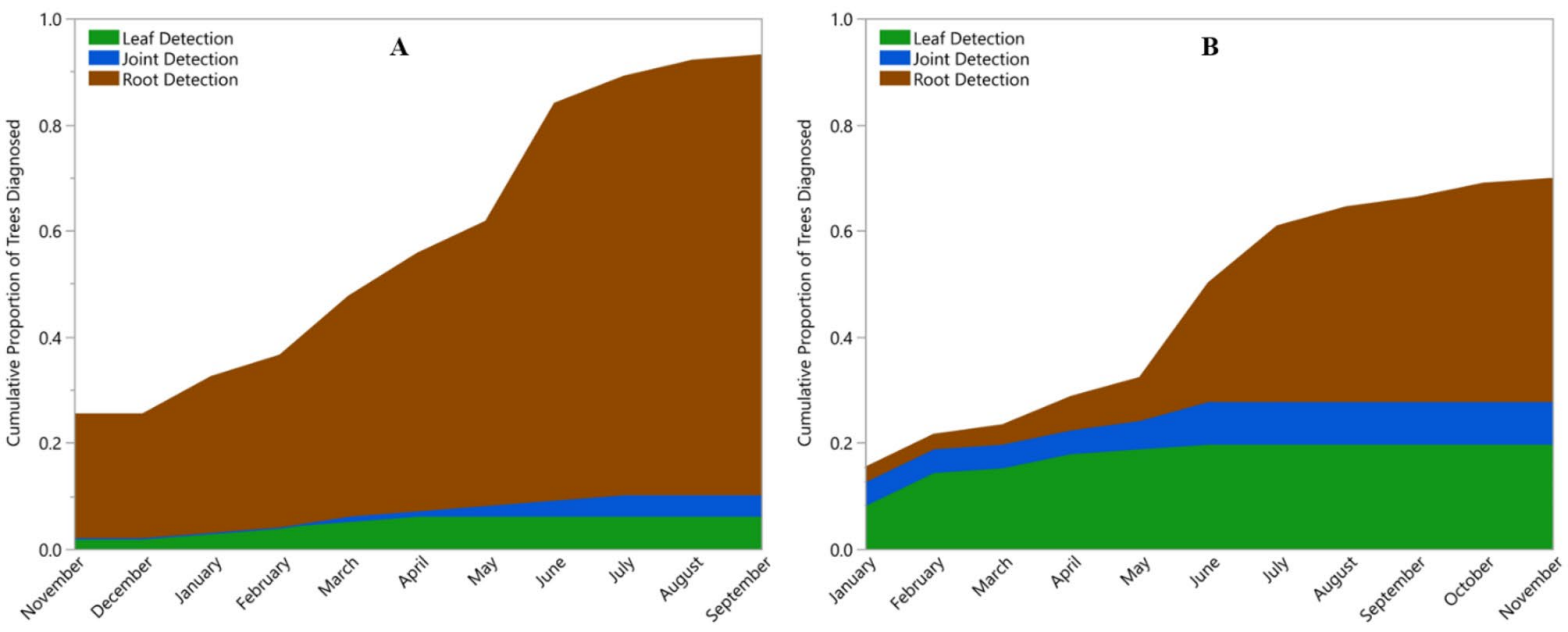
Cumulative proportion of *Citrus *trees diagnosed with Huanglongbing by real-time PCR detection of *Candidatus *Liberibacter asiaticus (CLas) in Florida (**A**) and Texas (**B**). The sample type by which each tree was diagnosed with HLB are indicated by color: Brown represents trees in which CLas was first detected in a root sample; Green represents trees in which CLas was first detected in a leaf sample; and Blue represents trees with simultaneous detection in roots and leaves.. Note, no samples were collected in December 2015 in Florida.

Patterns of CLas detection were quite similar among the sweet orange trees from Florida and the grapefruit trees from Texas. Using real-time PCR on DNA extracts from each sample, CLas was detected in 385 (19%) of the Florida samples and 410 (17%) of the Texas samples. This corresponded to diagnosis of 89 (90%) of the Florida trees and 82 (73%) of the Texas trees as infected with CLas by the end of the study (Fig. 2a,b). Although we did not continue recording data on symptomology, all trees in the first sampling period lacked visible symptoms. Despite this, we detected CLas in 25 Florida trees (23 among root samples and 2 among leaf samples) and 17 Texas trees (3 among root samples, 9 among leaf samples, and 5 in both sample types) in the first sample period.

### CLas titer

**Figure 3:**
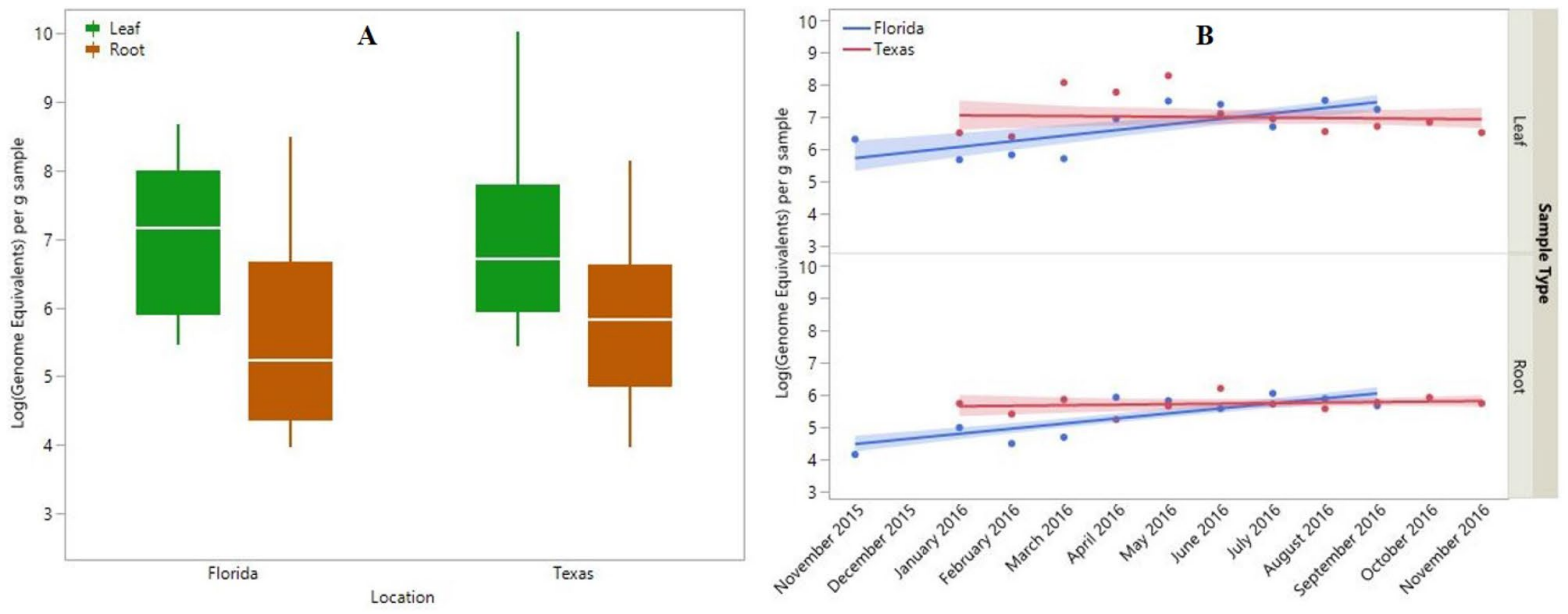
Bacterial titer per milligram sample, as estimated by the log of the number of *Candidatus* Liberibacter asiaticus genome equivalents, in *Citrus*. Panel A depicts the central tendency and variation in titer in the form of box plots for leaf (green) and root (brown) samples from Florida (left) and Texas (right). The data are presented as log_10_ values to better approximate a normal distribution. The horizontal line within the box represents the median number of genomes, the box spans the interquartile range (25% to 75% quantile), and the whiskers reach from these points to 1.5 times the interquartile range. Panel B depicts the average titer for each month in Florida (blue) and Texas (red). The lines represent the best fit linear regression and the shaded areas represent the 95% confidence intervals of the lines. Note, no samples were collected in December 2015 in Florida, and the September 2016 data points for Florida and Texas overlap.

Beyond detection, real-time PCR can be used to estimate bacterial titer (i.e., the estimated number of target molecules divided by the number of copies of the target in the CLas genome) in one gram of sample. The estimate of bacterial titer, Log_10_ (Genome Equivalents) per g, varied significantly between these groups (F_(41,753)_ = 8.38, p < 0.0001). Sample type and the interaction between sample location and sample period significantly impacted differences in titer while location alone had little impact. The average titer found among leaves was greater than that found among roots ($\overline{X}$± SE = 7.01 ± 0.09 and 5.67 ± 0.05 copies, respectively; Fig. 3a). Sample location, on its own, had little influence on bacterial titer with mean (± SE) genome equivalents in Florida and Texas of 5.86 ± 0.07 and 6.07 ± 0.06, respectively (Fig. 3a). However, the interaction between sample location and sample period revealed a significant and interesting pattern (F_(8,753)_ = 3.16, p < 0.0016; Fig. 3b). Bacterial titer increased through time in Florida (Leaf: F_(1,77)_ = 20.11, p < 0.0001; Root: F_(1,304)_ = 40.74, p < 0.0001) but remained stable in Texas (Leaf: F_(1,98)_ = 0.09, p = 0.76; Root: F_(1,308)_ = 0.42, p = 0.52).

### Frequency and consistency of detection

Sample type impacted the frequency with which the bacteria was detected. Contingency analysis among trees showed that leaf samples identified infected trees less often than expected whereas root samples diagnosed infected trees more often than expected by chance. In Florida, CLas was detected among leaf samples of 37 trees (42% of diagnosed trees) and from root samples of 88 trees (99% of diagnosed trees; χ^2^_(1)_ = 60.74, p < 0.0001). Similarly, in Texas CLas was detected among leaf samples of 37 trees (45% of diagnosed trees) and from root samples of 74 trees (90% of diagnosed trees: χ^2^_(1)_ = 24.91, p < 0.0001). In all, more than twice as many trees produced positive root samples than leaf samples.

Similar patterns were observed when individual samples, rather than trees, were compared. Contingency analysis of samples showed that leaf samples contained detectable CLas less often than expected whereas roots contained detectable CLas more often than expected. Of the 385 samples from which CLas was detected in Florida, 306 (79%) were root samples (χ^2^_(1)_ = 221.34, p < 0.0001). The same pattern held in Texas where 310 (76%) of the 410 CLas detections were found among roots (χ^2^_(1)_ = 172.43, p < 0.0001). Across all samples, there were more than three times as many positive root samples as leaf samples.

**Figure 4:**
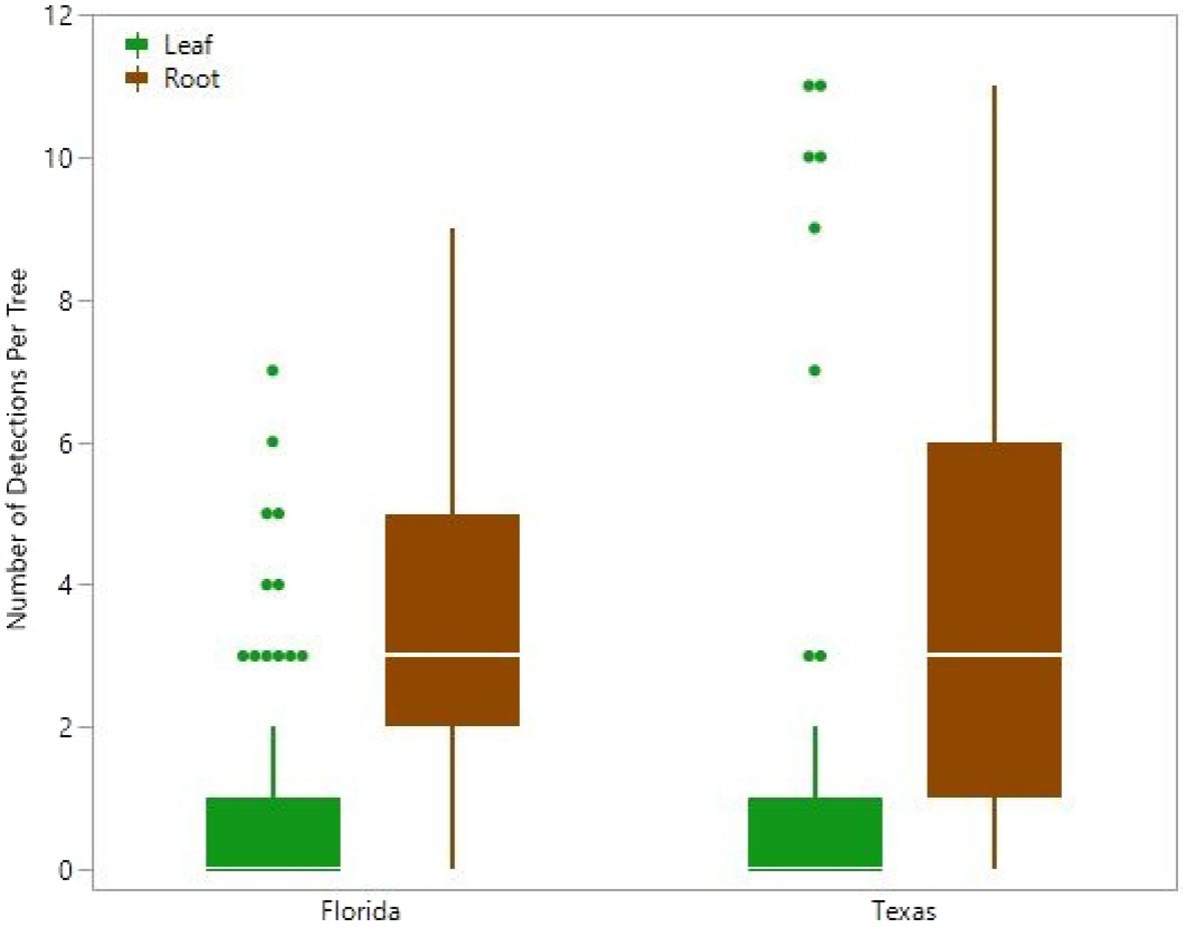
The consistency of detecting *Candidatus* Liberibacter asiaticus (CLas) within infected *Citrus* trees is shown by the number of detections per tree from monthly samples of trees infected with CLas. Green and brown box plots indicate leaf and root samples, respectively. The horizontal line within the box represents the median number of detections, the box represents the interquartile range (25% to 75% quantile), and whiskers reach from these points to 1.5 times the interquartile range and define statistical outliers, which are represented by dots.

The increased frequency of detection of CLas among root samples corresponded to an increase in the consistency of detection within individual trees (Fig. 4). Not only was CLas detected among roots more often than among leaves, but it was detected more consistently among roots as well. Each tree was sampled repeatedly resulting in ten and eleven sample periods per tree for Florida and Texas trees, respectively. The number of detections per tree, from trees found to contain CLas, was significantly greater for roots than leaves with an average of 3.4 detections per tree for roots and 0.89 for leaves from Florida trees (t_(176)_ = 9.72, p < 0.0001) and 3.8 for roots and 1.2 for leaves from Texas trees (t_(162)_ = 5.79, p < 0.0001; Fig. 4). Moreover, contingency analysis showed that the proportion of samples from which CLas was detected after the initial detection was significantly greater for root than leaf samples in both Florida (χ^2^_(1)_ = 225.9, p < 0.0001) and Texas (χ^2^_(1)_ = 172.43, p < 0.0001). Regardless of the sample type from which CLas was first detected, it was detected again in 15% of the subsequent leaf samples compared to 60% of the subsequent root samples in Florida. Similarly, after CLas was first detected in a Texas tree, 17% of the subsequent leaf samples detected it compared to 53% of the subsequent root samples.

### Timing of detection

Detection of CLas was achieved earlier with root samples than with leaf samples. In 79 Florida trees (89% of trees diagnosed as infected), CLas was first detected in root samples while in only six trees (7% of trees diagnosed as infected) were leaf samples the first to produce detectable CLas. Four Florida trees had detections of CLas in root and leaf samples simultaneously (Fig. 2a). In Texas, the first detection of CLas was from root samples in 51 trees (62% of diagnosed trees) and from leaf samples in 22 trees (27% of diagnosed trees). In nine Texas trees, CLas was detected simultaneously in roots and leaves (Fig. 2b). Contingency analysis found these differences to be statistically significant (χ^2^_(1)_ = 14.89, p < 0.0001).

**Figure 5:**
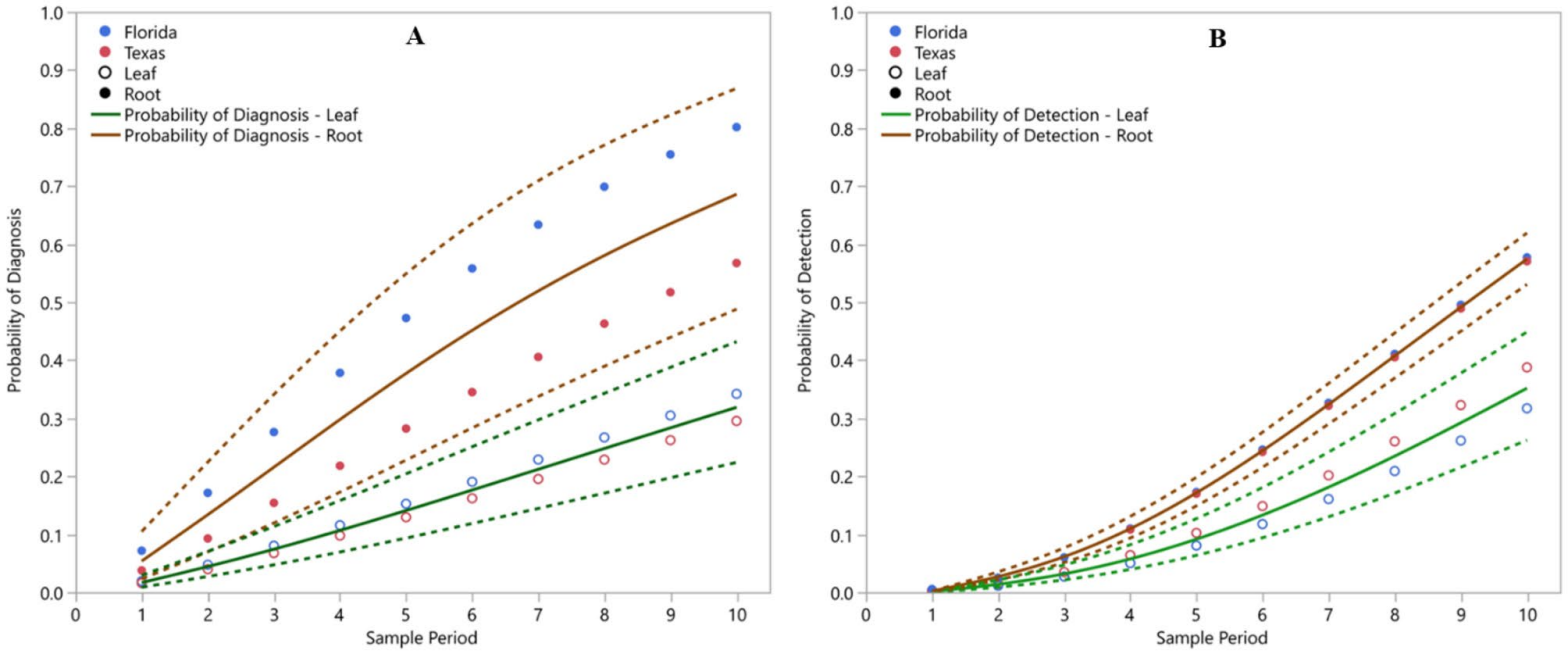
The probability functions for diagnosing trees with HLB (i.e., the first detection regardless of tissue) (**A**) and detecting *Candidatus* Liberibacter asiaticus among samples (i.e., including repeated detections) (**B**) using DNA extractions from *Citrus* leaves (solid green line) and roots (solid brown line). Dashed lines represent the upper and lower 95% confidence intervals of the estimate of the corresponding color. Geographic variation exists in these functions as depicted by probability estimates from Florida (blue circles) and Texas (red circles). While the estimated probability of detection from leaves (open circles) varies between Florida and Texas to a greater degree than do roots (closed circles), these differences are not statistically different from one another. Sample period represents the order in which the monthly samples were collected.

We compared the time until diagnosis (i.e., first detection of CLas regardless of sample type) for each tree using parametric survival analysis of leaf and root samples. The distribution of times to diagnosis were best fit by the Weibull distribution (AICc = 1695.80, BIC = 1715.89) and the fit was statistically significant (χ^2^_(3)_ = 84.03, p < 0.0001). Sample type (leaf or root) and location had significant impacts on time until diagnosis (χ^2^_(1)_ = 59.29, p < 0.0001 and χ^2^_(1)_ = 9.16, p = 0.0025, respectively). The interaction between sample type and location was not significant (χ^2^_(1)_ = 3.02, p = 0.08). Using these relationships, we estimated the probability of diagnosing trees with HLB from leaf samples and from root samples. The probability of diagnosing trees with HLB changed through time, as shown in Fig. 5a, and was significantly higher in roots than in leaves at all time points (p < 0.05 for each time point).

To evaluate the probability of detection from any given sample, we compared the time until detection for each tree using parametric survival analysis of leaf and root samples. The distribution of times to detection were best fit by the Weibull distribution (AICc = 5292.52, BIC = 5317.99) and the fit was statistically significant (χ^2^_(3)_ = 72.42, p < 0.0001). Using this model, a significant impact of sample type (leaf or root) on time until detection (χ^2^_(1)_ = 70.52, p < 0.0001) was found. However, neither location (Florida or Texas), nor the interaction between sample type and location had a significant impact on time until detection (χ^2^_(1)_ = 1.67, p = 0.17; χ^2^_(1)_ = 2.50, p = 0.11, respectively). Using these relationships, we estimated the probability of detecting CLas from leaf samples and from root samples. The probability of detecting CLas changed through time, as shown in Fig. 5b, but was significantly higher in roots than in leaves at all time points, from the beginning of the study through the end (p < 0.05 for each time point).

**Figure 6:**
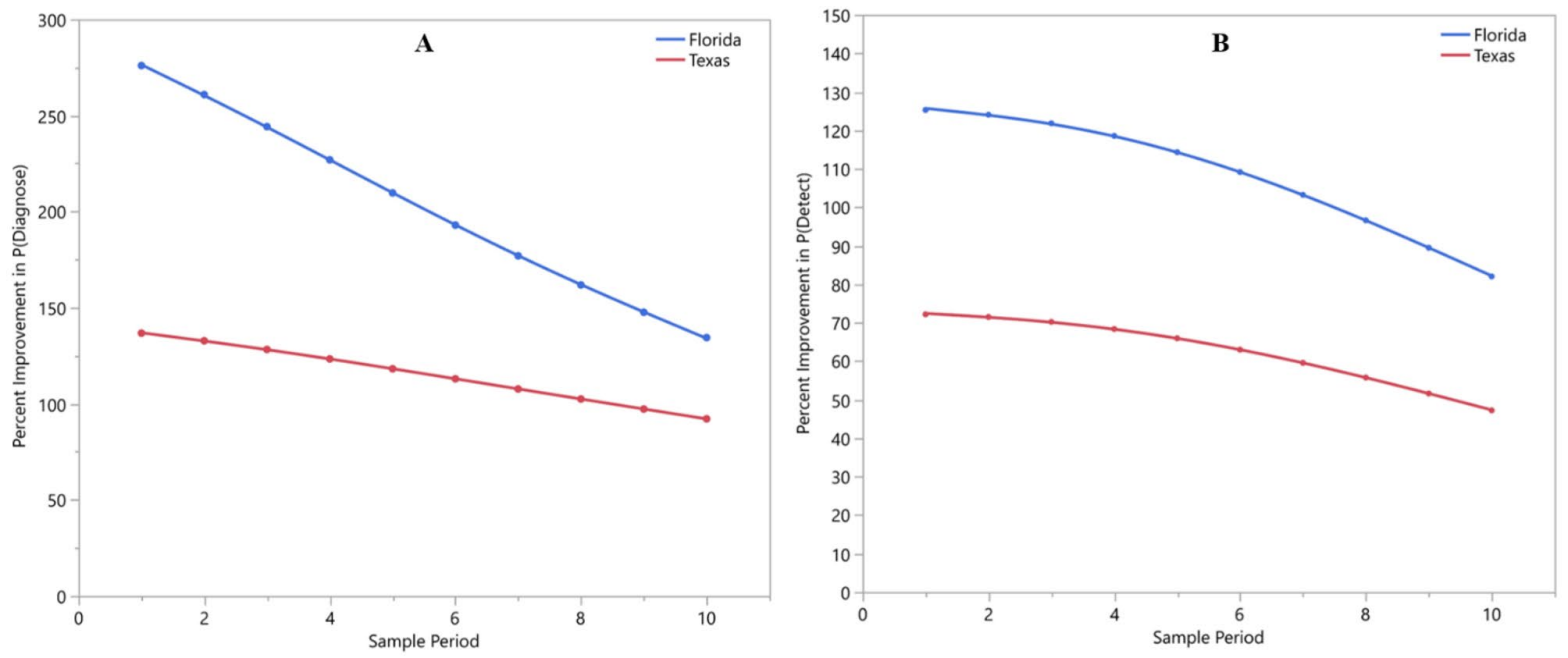
Percent improvement in the probability of diagnosing trees with HLB (i.e., the first detection regardless of tissue) (**A**) and detecting *Candidatus* Liberibacter asiaticus among samples (i.e., including repeated detections) (**B**) using DNA extractions from *Citrus* root samples over those from *Citrus* leaf samples. The percent improvement observed in Florida is shown in blue while that in Texas is shown in red. Sample period represents the order in which the monthly samples were collected.

**Figure 7:**
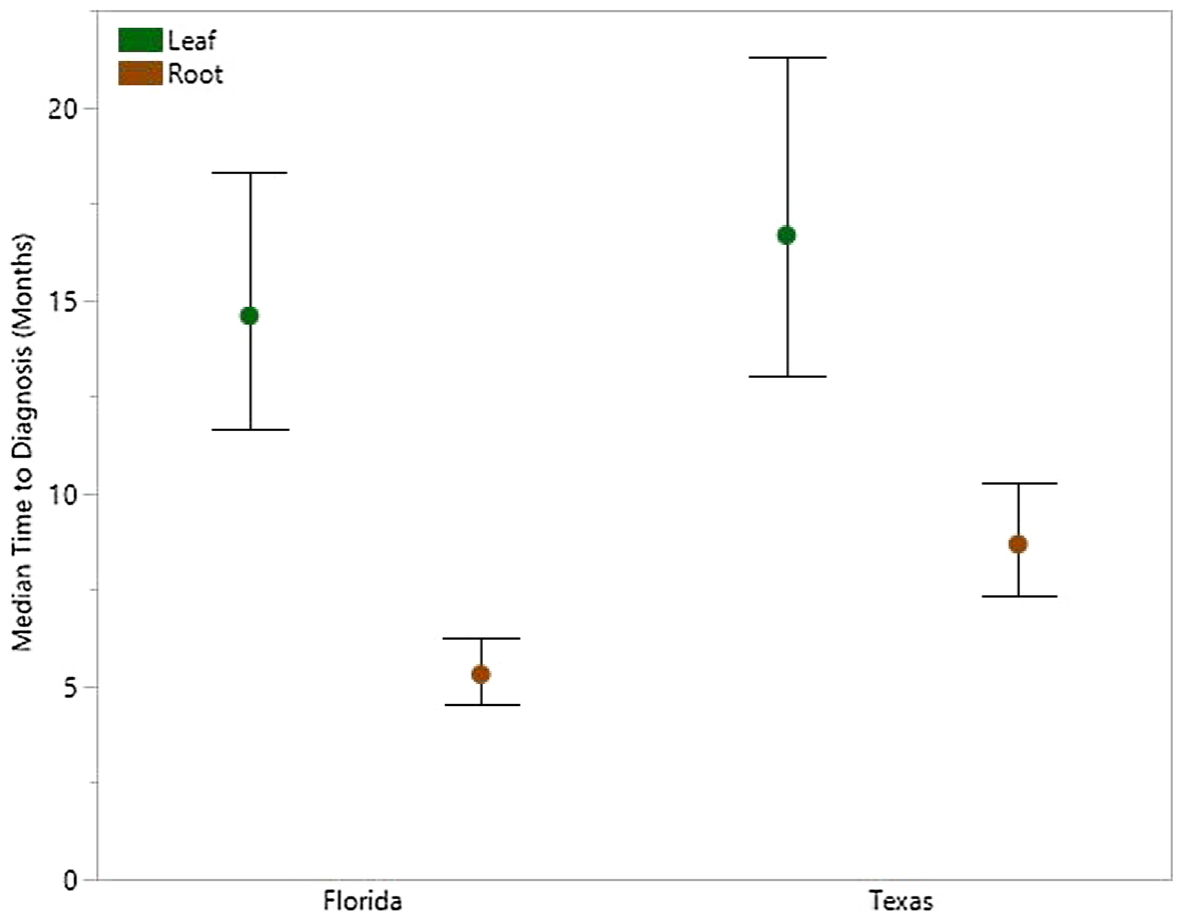
Earlier detection of *Candidatus* Liberibacter asiaticus in *Citrus* root samples is shown by the time to diagnosis of 50% of trees estimated by the survival model. The estimated median time (± 95% confidence intervals) to diagnosis trees is shown for Florida (left) and Texas (right) using leaf (green) and root (brown) samples.

The improvement in the probability of diagnosing trees and detecting CLas among samples gained through the use of root samples was greater in Florida than in Texas. Diagnosis of trees (i.e., first detections) improved by more 275% in Florida and 136% in Texas through the use of root samples. While still substantial, this improvement declined through time to 134% and 92%, respectively, by the end of the study (Fig. 6a). Similarly, the probability of detecting CLas among individual samples was improved through the use of roots by more than 125% in Florida and by nearly 75% in Texas. The improvement declined to 82% and 47% for Florida and Texas, respectively, over the duration of the study (Fig. 6b). Survival models estimated that 50% of the trees would be diagnosed 9 months earlier in Florida and 8 months earlier in Texas through the use of root samples (Fig. 7).

## Discussion

HLB poses a significant threat to citriculture around the world due to the lack of commercial *Citrus* cultivars tolerant or resistant to HLB. Currently available measures to limit the spread of HLB include control of the insect vector population, removal and destruction of infected trees, and the exclusive use of pathogen-free budwoods^40^. The utility of tree removal and destruction relies on rapid and accurate diagnostics.

Early detection of CLas has long been a goal of the *Citrus* community^31,32^. A number of researchers have focused efforts on improving the rate of detection using a wide variety of methods including: differences in spectra^41^ or polarization of light reflected from leaves using portable devices^42,43^ or satellite imagery^44^; the presence of CLas secreted proteins^45^; and the presence of salivary sheaths from Asian citrus psyllid (ACP)^46^. However, commercial growers have been hesitant to adopt methods not approved by regulators, and regulators require direct evidence of the pathogen’s presence (i.e., its genome).

The “gold standard” for detecting CLas is real-time PCR-based methods targeting various regions of CLas genome^30,35,47,48^. However, pre-symptomatic detection from leaf samples has proven difficult due to the uneven (patchy) distribution of CLas within the tree canopy^27,33^. This study was conducted to evaluate if diagnostic tests using *Citrus* root samples could improve upon early detection.

After collecting, isolating DNA, and using real-time PCR to test for the presence of CLas from 4,444 samples in Florida and Texas across 11 months, we found that most of the samples (82%) failed to detect CLas. However, the frequency of detection increased over time presumably as disease spread among these trees. By the end of the study, 90% of trees in Florida and 73% of trees in Texas had been diagnosed with HLB. Interestingly, we detected CLas in 42 trees that lacked visible symptoms at the beginning of the study. Most of these detections were among root samples (n = 31) as opposed to leaf samples (n = 16). However, 11 trees produced detections in leaves but not roots at this time point. One hypothesis is that these samples were taken prior to pathogen migration to the roots. Moreover, the relative dearth of such trees in Florida (2 trees), where the incidence of HLB is higher, compared to Texas (9 trees) would support such a hypothesis. However, such limited sample sizes make it difficult to differentiate from random chance. Regardless, these results indicate that both leaf- and root-based diagnostics can detect CLas prior to the initiation of symptoms but suggest that roots may be the more efficient sample for pre-symptomatic detection.

As reported previously^28^, CLas titers were higher in leaves than roots. This pattern appears to be widespread as we observed little difference between Florida and Texas samples. However, an interesting pattern emerged through time and across space. CLas titer increased through time in Florida, but not in Texas (Fig. 3b). Several hypotheses could explain this pattern. First, sampling began earliest in Florida and may have captured the early invasion of CLas into these trees. Captured early enough, one would expect to see titer increase within individual plants while incidence increases among plants. This hypothesis would suggest that titer should level off in Florida as most trees become infected. This hypothesis seems unlikely as more trees were diagnosed on the first sampling period and a greater proportion of trees became infected by the end of the study in Florida than in Texas (Fig. 2a,b). Alternatively, psyllid-driven re-inoculation could explain the increase in titer through time in Florida while lower psyllid population sizes in Texas explain the lack of this increase. Finally, CLas titer may respond to environmental variables. There is substantial evidence from both greenhouse^21^ and field studies^49,50^ to support this conclusion. This hypothesis would suggest that the variation about the regression line the data from Texas is not mere error, but rather a non-linear relationship. There is insufficient data at this time to distinguish between these hypotheses.

Among the trees diagnosed with HLB, there was a dramatic difference in the frequency with which we detected CLas in each sample type. More than twice as many trees diagnosed with CLas produced positive root detections than produced positive leaf detections and more than three times as many root samples produced positive detections of CLas than leaf samples. The results were remarkably similar between sweet orange trees in Florida and grapefruit trees in Texas suggesting the result is general among *Citrus* varieties and across environments.

The increased frequency of detections using root samples was mirrored within individual trees. The average number of detections per tree was over three times higher in root samples than in leaf samples. This improvement in consistency of detection may be due to the even distribution of CLas within roots and the patchy distribution of CLas within the canopy^27,33^. Louzada et al.^33^ found 96% of horizontally growing roots just under the soil surface contained detectable quantities of CLas. It is possible that the consistency of detection in the canopy improves through time as more leaves become infected (see below).

By asking which sample (leaf or root) provided earliest evidence of CLas infections for each tree, the current study found that CLas was first detected in root samples for more than 75% of the infected trees. Moreover, survival analysis documented significantly higher probabilities of diagnosis (Fig. 5a) and detection (Fig. 5b) at each sample period. These results support the hypothesis posed by Louzada et al.^33^ and Park et al.^35^.

Although geographical location did not have a statistically significant impact on the probability of detecting CLas nor a significant interaction effect with sample type, interesting patterns emerged when comparing the impact of location between leaf and root samples. The probability of diagnosing trees with HLB among root samples, but not leaf samples, varied between Florida and Texas. Florida had higher probabilities of diagnosing with roots than Texas (Fig. 5a). Conversely, the probability of detecting CLas among leaf samples, but not root samples, varied between Florida and Texas. In this case, Texas leaf samples had a higher probability of detecting CLas than those from Florida (Fig. 5b). Additionally, the probability of diagnosing trees with HLB and detecting CLas within samples changed over time. For both sample types, these probabilities increased with time. However, the degree of improvement gained by using root samples declined and differed between Florida and Texas (Fig. 6a,b). The difference in rates of improvement were likely an artifact due to the fewer number of trees remaining to be detected by root sampling. However, it is possible that canopy inoculation rate increased as incidence rate increased among surrounding trees, that establishment rate increased as tree health declined, or that transport of CLas from roots to leaves increased as infection spread throughout the root system.

The TXCChlb- and HLBaspr-based assay systems produce highly consistent results using leaf samples (data not shown). A small number of discrepancies, however, do occur among samples with Cq values near the Cq cutoff (e.g. 36.5 < Cq < 37) due to the slightly lower amplification efficiency of TXCChlb (data not shown). Despite this possibility of underestimating detections using TXCChlb, our marker for roots detections, the improved probability of diagnosing trees with HLB using root samples was apparent. This improvement was likely due to the higher consistency of detection and more even distribution of CLas within roots^33^. We suspect that further improvement would be seen through the use of root samples and assays built on CLas genic regions with higher amplification efficiencies such as the *nrdB* gene, which has five copies in the CLas genome^51^. Adaptation of such markers to root samples would be beneficial as survivorship models suggest that root-based diagnostics may alert growers to the presence of CLas within their orchards months earlier than leaf-based diagnostics.

Current management strategies generally rely on visual inspection of the tree canopy for distinct HLB symptoms followed by collection of symptomatic leaves for HLB diagnostic test by real-time PCR in the lab. This approach effectively offers confirmation of visual hypotheses. As previously reported by Coletta-Filho et al.^26^ and Lee et al.^17^, HLB spread from pre-symptomatic trees is likely taking place in the field. Pre-symptomatic diagnosis offers a route to limiting spread by triggering earlier deployment of HLB control measures. The current study clearly indicates the benefit of using fibrous root samples for HLB diagnosis. In regions where HLB and/or its insect vector have not yet been reported, or in the areas where HLB incidence is localized, surveying the trees at the edge of the field^52,53^ using root-based diagnostics, may provide growers early insight into disease presence even in the absence of visible symptoms.
